# Reducing conflict and containment rates on acute psychiatric wards: The Safewards cluster randomised controlled trial

**DOI:** 10.1016/j.ijnurstu.2015.05.001

**Published:** 2015-09

**Authors:** Len Bowers, Karen James, Alan Quirk, Alan Simpson, SUGAR, Duncan Stewart, John Hodsoll

**Affiliations:** a Institute of Psychiatry, King's College London, De Crespigny Park, London SE5 8AF, United Kingdom; b Royal College of Psychiatrists, 21 Prescot Street, London E1 8BB, United Kingdom; c City University London, Northampton Square, London EC1V 0HB, United Kingdom

**Keywords:** Absconding, Inpatient, Psychiatry, Rapid tranquillisation, Restraint, Seclusion, Self harm, Special observation, Violence

## Abstract

**Background:**

Acute psychiatric wards manage patients whose actions may threaten safety (conflict). Staff act to avert or minimise harm (containment). The Safewards model enabled the identification of ten interventions to reduce the frequency of both.

**Objective:**

To test the efficacy of these interventions.

**Design:**

A pragmatic cluster randomised controlled trial with psychiatric hospitals and wards as the units of randomisation. The main outcomes were rates of conflict and containment.

**Participants:**

Staff and patients in 31 randomly chosen wards at 15 randomly chosen hospitals.

**Results:**

For shifts with conflict or containment incidents, the experimental condition reduced the rate of conflict events by 15% (95% CI 5.6–23.7%) relative to the control intervention. The rate of containment events for the experimental intervention was reduced by 26.4% (95% CI 9.9–34.3%).

**Conclusions:**

Simple interventions aiming to improve staff relationships with patients can reduce the frequency of conflict and containment.

**Trial registration:**

IRSCTN38001825.

**What is already known about the topic?**

• There are no previous RCTs of interventions to reduce conflict or containment as a whole. Even for individual conflict behaviours or containment events (e.g. violence alone or seclusion alone) very few RCTs have been undertaken.
• There are substantially more before and after studies of interventions in practice, which are mostly local and without controls, and a large quantity of observational, longitudinal and descriptive studies.
• Narrative reviews are available, particularly for violence, and for seclusion and mechanical restraint. They suggest that conflict and containment rates can be influenced by staff behaviour, but the evidence is generally weak and of poor quality.
• What evidence exists has been assembled into the Safewards Model, which underlies the interventions used in this trial.

**What this paper adds**

• Both conflict and containment overall can be reduced, making wards safer and less coercive environments for patients and staff.
• The underlying Safewards Model is supported and should now be subjected to further tests.
• The ten easy Safewards interventions should be implemented in practice.

Acute psychiatric wards provide limited duration care to people in acute states of disturbance and distress. Once admitted patients may exhibit a number of different difficult and risky behaviours, including verbal aggression, attempts to abscond, self-harm, refusal to eat or drink, aggression to objects or people. A range of different methods are used by nursing staff either to prevent these behaviours from occurring, or ameliorate their outcomes, including the use of extra tranquillising medication, special observation by staff, manual restraint and seclusion. We refer to the behaviours posing a risk to patients or those around them as ‘conflict’, and the actions of staff to manage them as ‘containment’. Treating these behaviours collectively is justifiable because the different conflict behaviours (aggression, self-harm, substance/alcohol use etc.) correlate strongly within patients (Bowers et al., 2005) and within wards (Bowers, 2009). Patients who engage in high rates of one type of conflict behaviour are more likely to engage in others, and wards with high rates of one type of conflict behaviour are more likely to have high rates of others. The same is the case for different containment items, both for patients and wards.

Despite the link between different conflict and containment events, and the connection between conflict and containment themselves, most current methods for making psychiatric wards safer places focus on just one or two types of these events. Training courses for staff in the prevention and management of violence are the most commonly used intervention. These contain basic de-escalation skills and manual restraint training (Lee et al., 2001). The ‘six core strategies’ are another approach, in this case targeted at reducing the use of seclusion and mechanical restraint (Huckshorn, 2005) and include: senior management commitment to change, using audit to inform practice, workforce training, use of assessment tools, patient involvement, and debriefing techniques. A third current method aims to reduce violence through short term (shift by shift or day by day) risk assessment based on statistically verified indicators (Abderhalden et al., 2004). The majority of evaluations of these and other methods have been by natural experiment with official statistics as the outcome measure (*n* = 103 studies). Results have been variable (Stewart et al., 2010), the use of any control group rare (*n* = 20 studies), the possibility of substitution of one form of containment by another seldom assessed, the issue of missing data ignored, and the number of previous randomised trials very small (*n* = 5). Publication bias in relation to the high numbers of natural experiments is likely. None of these methods for making psychiatric wards safer provide a comprehensive model explaining the causes of conflict and containment events, nor do they aim to reduce conflict and containment as a whole. All figures for the numbers of studies refer to a cross topic review of the whole conflict and containment literature, ultimate consisting of 1177 papers and conducted by the authors’ research group.

Previous research has shown highly variable rates of conflict and containment on different wards, not explicable solely in terms of the patients admitted (Bowers, 2009). The cross topic literature review referred to above (Bowers et al., 2010; Dack et al., 2013; James et al., 2012; Owiti and Bowers, 2011; Papadopoulos et al., 2012a,b; Stewart and Bowers, 2011; Stewart et al., 2009; Van Der Merwe et al., 2013) led to the development of the Safewards Model (Bowers, 2014). This model explains variable rates of conflict and containment and identifies a large number of ‘staff modifiers’: aspects of staff actions that can impact on the likelihood of conflict or containment incidents. The model enabled the creation of a list of interventions that could enhance the staff modifiers and thereby reduce conflict and containment rates. As the focus of potential interventions in the Safewards trial was the nursing team as a whole and generalised aspects of their attitudes and behaviour towards patients, wards had to be the unit of randomisation. The list of potential interventions was scored by the research team for feasibility and impact, resulting in a short list of 30 that were taken to consultations with panels of expert nurses, service users and carers (Simpson et al., 2014). The top 16 interventions went forward to a pilot study on four wards, and were subsequently reduced, consolidated and improved into a package of ten interventions for use in a full scale cluster randomised controlled trial.

## 1 Materials and methods

### 1.1 Objective

We aimed to evaluate the efficacy of a complex intervention (Safewards), targeted at nursing staff, to reduce conflict and containment rates at the level of acute psychiatric wards.

### 1.2 Participants

The study comprised 31 psychiatric wards at 15 hospitals within 100 km of central London and in 9 NHS Trusts. Inclusion criteria were acute psychiatric wards for adults of any gender. Wards were excluded if they had a specialist function, had planned major changes, or where two or more of the following criteria were met: no permanent ward manager in post, a locum consultant solely responsible for inpatient care, >30% nursing staff vacancy rate. Willing nurses and healthcare assistants working on the selected wards were included, with 564 staff (88% of the possible total) giving their consent. Non-consenting staff were free not to submit outcome data, questionnaires, or participate in the interventions. Signed consent on behalf of patients was given by Trust CEOs, although no data was collected from patients and all research interventions were with staff. Directors of Nursing and Medical Directors also approved the study prior to access being granted. National Health Service ethical approval was secured (11/LO/0798).

### 1.3 Interventions

Wards in the experimental condition implemented a package of ten ‘Safewards’ interventions: (1) mutually agreed and publicised standards of behaviour by and for patients and staff; (2) short advisory statements (called ‘soft words’) on handling flashpoints, hung in the nursing office and changed every few days; (3) a de-escalation model used by the best de-escalator on the staff (as elected by the ward concerned) to expand the skills of the remaining ward staff; (4) a requirement to say something good about each patient at nursing shift handover; (5) scanning for the potential bad news a patient might receive from friends, relatives or staff, and intervening promptly to talk it through; (6) structured, shared, innocuous, personal information between staff and patients (e.g. music preferences, favourite films and sports, etc.) via a ‘know each other’ folder kept in the patients day room; (7) a regular patient meeting to bolster, formalise and intensify inter-patient support; (8) a crate of distraction and sensory modulation tools to use with agitated patients (stress toys, mp3 players with soothing music, light displays, textured blankets, etc.); (9) reassuring explanations to all patients following potentially frightening incidents; and (10) a display of positive messages about the ward from discharged patients. Interventions therefore occurred at the cluster level, as they were collective endeavours of the nursing team, or visible to everyone. Full descriptions of these interventions coupled with training videos are freely available online (www.safewards.net). Wards in the control condition implemented a package of interventions directed at improving staff physical health: a desk exercises poster in ward office; pedometer based competitions; supplies of healthy snacks; diet assessment and individualised feedback; health and exercise magazines supplied regularly to the staff office; health promotion literature; linkages to local sports and exercise facilities. Improvement in physical health was predicted by the Safewards Model to have no impact on conflict and containment. This arm of the study therefore controlled for both researcher attention and participant expectancy. All wards and their staff in both arms were primed to expect reductions in conflict and containment rates. Staff on the control wards were told that improvements in their own physical health would lead to them delivering nursing care more effectively, and thereby reduce conflict and containment.

### 1.4 Outcomes

The primary outcomes were rates of total conflict and rates of total containment as measured by the Patient-staff Conflict Checklist (PCC) (Bowers et al., 2005). This single sheet paper form was completed by the nurse in charge at the end of every nursing shift, and logs the frequency of 22 conflict events (verbal aggression, suicide attempts, alcohol use, attempted absconding, etc.) and 8 uses of containment (coerced medication, seclusion, restraint, special observation, etc.). These events are recorded at the level of the shift, not individual patient, for example a PCC may record 3 verbal abuse events, 1 attempted abscond and 1 self-harm event, but does not record which patients were responsible for those events, or whether one patient was responsible for them all. A total conflict score is obtained by summing the number of conflict incidents during the shift, and a total containment score by summing the number of containment events. The tool has been demonstrated to be reliable (Bowers et al., 2006) and valid (Bowers et al., 2005) and is accompanied by a handbook, carefully devised operational definitions, and brief structured training. Its associations with ward features, staffing provision, patient characteristics, physical environment, routines, surrounding local community service provision, and change over time in relation to local policy changes and other events, have been thoroughly explored in some of the largest observational studies conducted into acute psychiatry to date (Bowers et al., 2007a,b). Secondary outcomes were the Attitude to Personality Disorder Questionnaire (Bowers and Allan, 2006), the Self-harm Antipathy scale (Patterson et al., 2007), the Ward Atmosphere Scale (programme clarity, and order and organisation subscales) (Moos, 1974); and the SF-36v2, a short form health survey (Ware et al., 2002). These additional scales were those best representative of the types of changes in staff predicted by the Safewards Model to be associated with changes in rates of conflict and containment. In addition the SF-36v2 was included to assess the impact of the control interventions. Fidelity was measured by a simple checklist completed by Research Assistants on every visit to the wards and by a participant end of study questionnaire.

### 1.5 Sample size

The required sample size was based on the data from the City-128 study (Bowers, 2009), extrapolated to a full trial scenario through simulation, powered for two primary outcomes by Bonferroni adjustment. Conflict and containment have a complex and partial relationship, therefore these two types of events need to be assessed independently. The model for event counts (conflict or containment) was Poisson based with offset for number of beds per ward and random effects of ward nested within hospital to account for clustering due to paired randomisation of wards within hospital and repeated measures within ward. A conservative figure of 13 beds per ward was assumed, with 2 wards per hospital and 10 hospitals participating. For periods of 30 days (three shifts per day, 90 nursing shifts in total) in each phase the model predicted 97.9% power for conflict events and 93.7% power for containment events. The calculations allowed for a modest 20% decreases in target events commensurate with those obtained in our previous before and after trials (Bowers et al., 2003, 2006). In order to allow for potential ward drop outs from the trial, a target of 15 hospitals (30 wards) was set for the study.

### 1.6 Randomisation and masking

**Fig. 1:**
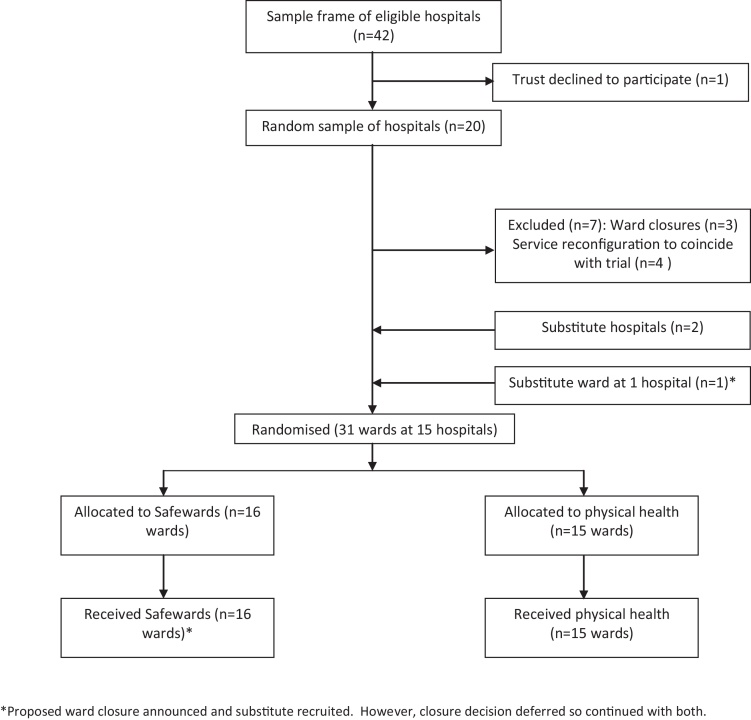
Trial profile.

All hospital sites within 100 km of central London and with at least two eligible wards were identified. Three random selections were made: (i) hospitals, (ii) two wards at each hospital, (iii) allocation to experimental or control (Fig. 1). In each case simple randomisation was used for the selection process by the designated staff member at King's College Clinical Trials Unit. At one hospital site with three eligible wards there was uncertainty as one ward of two was potentially going to close. All three wards were therefore recruited and the two wards under threat of closure were randomised to the same experimental condition. No ward closed during the study, resulting in a total sample size of 31 wards. No wards dropped out from the study. All randomisation was independent of the researchers and trial statistician. Throughout the study, wards and their staff were blind as to which package of interventions were the experimental or control condition, each of which were given neutral titles and were described as likely to reduce conflict and containment rates. Additionally neither the staff nor the research assistants working with them knew which intervention package would be applied on which ward until two weeks before its introduction, so that baseline data could not be biased.

### 1.7 Procedure

Once recruited, ward staff were trained in the use of the PCC and collection of data on the primary outcome continued throughout the study (see Fig. 1). Baseline data were collected for eight weeks, and wards then had a further eight weeks to implement their allocated package of interventions. They then continued using the interventions for a further eight weeks. Secondary outcome questionnaires were collected during the baseline period and repeated during the outcome period, and were distributed to consenting staff via internal mail or in person. All wards were visited 2–3 times a week throughout the study by researchers, who picked up and delivered questionnaires, encouraged participation, liased with the team to plan introduction of the interventions, and answered any questions. The trial is registered, number IRSCTN38001825, and an independently chaired Trial Steering Committee had oversight of the project.

### 1.8 Statistical analysis

The statistical analysis plan was agreed with the Trial Steering Committee before data collection began. Data were analysed using the MCMCglmm package in R 2.15 (Hadfield, 2010). The primary outcome was counts of conflict and containment events by ward shift (am, pm and night) collected over the course of the study phases; baseline, implementation and outcome. As the distribution of events in the data had an excess of zero counts (shifts with no events) we chose to model the data with a Poisson hurdle mixed model. Count data is generally modelled with Poisson distribution; however, it is often the case that there is greater heterogeneity in the data than expected by the Poisson model which can be manifest in numbers of zero events, as here. A hurdle model is a two-part model consisting of a binary response and count regression model, which has previously been useful in research on addictions (Atkins et al., 2013) and elsewhere. The first part of the model, the hurdle is based on the binomial distribution and describes the risk of a conflict or containment incident occurring (and therefore accounts for the zero events). If an event does occur, i.e. once the hurdle has been crossed, a zero-truncated Poisson distribution models the number of events occurring. The dependent variable was the count of incidents (conflict or containment) per shift. Covariates were study phase (baseline, implementation, and outcome), treatment (Safewards intervention or control), time of shift (am, pm or night), day in study phase (centred) and the log of the number of beds per shift to be used as an offset. An interaction between outcome phase and treatment condition gave the primary treatment difference at for the outcome phase. As the number of beds differed per ward the offset allows predicted rates of events per patient bed. Rate ratio estimates of the treatment effect are presented together with the 95% Bayesian credible interval and associated p statistic. There were high rates of missing data in the study, and consequently we took a range of pragmatic approaches to assess the robustness of our results, under both missing at random (MAR) and missing not at random (MNAR) assumptions (White et al., 2011). Further details of data analyses are in the supplementary information (SI).

### 1.9 Role of the funding source

The sponsor of the trial had no role in trial design, data collection, data analysis, interpretation, or writing of the report. The corresponding author had full access to all the data in the trial and had final responsibility for the decision to submit for publication.

## 2 Results

Of the 31 wards at 15 hospitals recruited to the study, 16 were assigned to the Safewards intervention package, and 15 to the physical health package. The mean number of beds per ward was 19 (SD 3.96) and most were generic acute wards (*n* = 24) with three triage/assessment wards and four psychiatric intensive care units. The majority served both male and female patients (*n* = 16) with ten serving men and five women only. The modal age group of the participating staff was 40–49 years (33.7%) a minority were white British (28.4%) and most were female (59.4%), all being typical of nursing staff working in acute psychiatry in the south east of England (Bowers et al., 2008a,b). There was no significant difference in ward type, gender served, staff age gender or ethnicity, between the experimental and control groups.

No wards dropped out of the study once recruited. However, the response rate for the primary outcome was less than expected, with less than 50% of PCC forms returned in the outcome phase. There was a range of return rates with some wards providing very high return rates and others with very low rates. However, the rates of missing data were approximately the same in the experimental and control conditions (see Table 3 and below) and we present a comprehensive investigation into possible biases due to missing data.

### 2.1 Primary outcome

Table 1 shows the baseline measures and Table 2 shows the rate ratios for the mean effect of treatment on the primary outcomes for the probability and rate of events. Relative to the control intervention, when conflict events occurred the Safewards intervention reduced the rate of conflict events by 15.0% (95% CI 5.6–23.7%). Similarly, when containment events occurred the rate of containment events for ward shifts with events was reduced by 26.4% (95% CI 9.9–34.3%). There were no significant differences in the rates of zero event shifts for conflict or containment.

### 2.2 Missing data and sensitivity analysis

Table 3 shows the number and percentage of missing observations by experimental phase and experimental condition. Baseline rates of missing data were relatively high at 36% for the control and 40% for the experimental condition. Although the increase in missingness was greater in the control than the Safewards condition, this difference was only present as a weak non-significant effect (OR – 0.87, 95% CI 0.74–1.03). There were no other predictors of missingness than already included in the primary analysis model.

Excluding wards from the analysis with high rates of missing data in the outcome phase had little impact on the treatment effect (for both the rate ratio and hurdle). Similarly, excluding hospitals with wards that did not comply with the protocol or that had operational difficulties did not effect the results. In all cases the direction and magnitude of the effect was approximately the same (Table 4, SI).

In the first imputation strategy we assumed all missing observations had zero events. In contrast for the second strategy we assumed that all missing shift reports were due to higher than average event rates. Neither of these strategies changed the direction of the treatment effect, rate ratios were 0.85–0.9 for conflict events and 0.76–0.9 for containment. This was the case even if missing observations were imputed to have absurd rates of events, e.g. over 100 conflict and containment events in 1 shift (Table 5, SI).

Next we assumed that missingness mechanisms were different in the two trial arms (despite no overall difference in the rates, Table 3) by imputing observations that were missing in addition to the baseline rate. Firstly, we considered that missing observations in control arm had the same rate of events as the present observations but those in the experimental condition were associated with increased rates of events. For conflict an increase of 2 events per shift was needed to reduce the rate ratio to 1 (RR for no effect, see Table 6, SI). For containment, a mean increase of 1 per missing shift was necessary. Given the large amount of missing data imputed, the need for a relatively large increase in event rates to change the direction of the experimental effect suggests our findings are robust.

### 2.3 Secondary outcomes

For the secondary outcomes, treatment effects (also in Table 2) showed no difference between the control and Safewards intervention for the Ward Atmosphere Scale, Self-Harm Antipathy Scale, Attitudes to Personality Disorder Questionnaire. On the SF-36 scale there was no difference in experimental group for the mental health measure, but there was an effect of group on physical health. The control group staff showed a 1.85 point (95% CI: 0.003–3.704) greater improvement in physical health than the Safewards intervention.

### 2.4 Fidelity

Assessing fidelity to the interventions was intrinsically difficult. The final target of most interventions was to organically change interactions between patients and between staff and patients. Detailed, intensive and laborious structured observation would have been necessary to capture this. As an alternative, researchers completed a checklist on every ward visit (2–3 times a week) scoring the presence of visible evidence of the use of each of the interventions, with a different checklist being used for the experimental and control wards. Each of the interventions provided differing degrees of visible evidence, some provided none. Often the fidelity score reflected evidence that was on display, rather than the degree to which staff engaged with and used the displayed material. Other scores were dependent on staff records generated during implementation of the interventions which were checked and rated by the researchers. Evidence for the control interventions was much easier to observe than for the experimental package. Fidelity checklist scores were converted into percentage implementation scores by regarding the maximum score as 100% and the minimum score as 0%. The mean fidelity to the experimental intervention by ward during the outcome period was 38% (SD 8, range 27–54%, *n* = 271) and for the control intervention 90% (SD 9, range 69–99%, *n* = 209). Examination of scores over time for the experimental wards showed a linear increasing trend from the start of the implementation period at 0% through to the end of the outcome period at 50%. Fidelity was also assessed via the completion of an end of study questionnaire by participants, in which they were asked whether they used each of the interventions. This was also converted into percentage fidelity scores by ward. Mean fidelity to the experimental intervention by ward by this measure was 89% (SD 11, range 62–100%, *n* = 79) and for the control intervention 73% (SD 19, range 39–100%, *n* = 74).

### 2.5 Credibility of the control intervention, efficacy of blinding, and contamination

Both control and experimental ward staff were provided with a rationale as to why their interventions should reduce conflict and containment, and that the researchers were interested to discover which set of interventions worked best. Via the ‘end of study’ questionnaire participants were asked whether they thought they were in the experimental or the control group. The majority in both groups thought they were in the experimental group, but that proportion was higher in the experimental group (88% vs. 74%). Comments during completion indicated that the question was often not understood, or was interpreted as ‘I took part in an experiment therefore I must have been in the experimental group’. Managers and staff were asked not to discuss the interventions in use on their own ward with people working on the ward in the opposing arm of the study. However via the ‘end of study’ questionnaire three quarters (116/147, 79%) admitted to engaging in such discussions.

## 3 Discussion

A large scale cluster RCT was conducted over a three month period in fifteen hospitals in and around London, with the aim of testing a package of interventions to increase safety and reduce coercion. The trial was a complex undertaking, requiring a large number of research staff operating across multiple sites, with considerable planning and organisation. Completion was dependent on the support and willingness of busy nursing staff to engage with the trial and undertake new and additional activities. Nevertheless, no ward dropped out from the study, and the trial intervention proved to be effective in reducing both conflict and containment.

There have been no previous randomised controlled trials of interventions to reduce conflict and containment together and across all types of event. The trials that have taken place have been restricted to one or two items, usually violent incidents, or seclusion and mechanical restraint (Abderhalden et al., 2008; Prescott et al., 2007). They have been mostly limited to samples of wards at single hospitals (Van De Sande et al., 2011), inactive treatment as usual controls (Putkonen et al., 2013), or simply report change over time without any control comparison at all (Pollard et al., 2007). The most stringent control over potential bias or other threats to validity such as trial registration, binding commitments to main outcomes and analysis plans in advance of results, trial steering committee management, fully independent randomisation, blinded independent analysis, blinding of participants, an active control intervention, outcome measures of proven validity and reliability, do not appear to have been utilised in any previous study in this field. All previous studies relied upon official incident reports or forms that were only completed by staff once incidents had occurred. Using this method there never appears to be any missing data, and rates cannot be assessed for bias. Using the PCC meant that missing data rates became visible and potential bias could be assessed. The Safewards trial thus represents a new step in the rigour, scale and scope of research into patient and staff safety in inpatient psychiatry.

The trial did yield an undesirably high level of missing data, despite the regular visits of researchers to the wards. Not all staff consented to the study, therefore on some occasions the nurse in charge did not feel obliged to complete the end of shift PCC. On other occasions staff were busy with other matters, the close of a nursing shift being when many reports are assembled and messages passed on in handover to the oncoming team of nurses. This moment both increases the likelihood of accuracy of a PCC (information is already being assembled) and decreases the likelihood it will be completed. The real issue with respect to the trial is whether the data were missing at random, and if not, did any bias undermine the findings? We believe our sensitivity analysis strongly supports the interpretation that this was not the case, however no such analysis can ever completely substitute for a full dataset. Some degree of caution must therefore be expressed about our findings.

It is hard to be certain about the degree of implementation of the research interventions. The ‘end of study’ questionnaires were positive, but the numbers of these questionnaires represented a low response rate and the chance of response bias towards exaggerated fidelity high. Objective observational measures taken by the researchers showed more modest implementation rates, however these did climb steadily over time. Acute psychiatric wards are busy and chaotic environments, with constant patient turnover and large teams of nurses and others working on a shift system seven days a week (Cleary et al., 2011). Asking them to implement ten interventions, however small, across the whole team and within an eight week period was a huge demand. A longer time period clearly would have allowed a greater degree of intervention implementation. As it was, implementation stretched out of the implementation phase of the research and continued, intensifying throughout the outcome phase.

A fall in the rate of conflict also occurred on the control wards, albeit not as large at that on the experimental wards. This may represent a true placebo effect, the result of expectancy of a beneficial change, but in that case containment should also have shown some decrease. The pedometer intervention on the control wards may have increased nurses’ movements around the ward, making them more available to patients, or enabling early intervention to avert conflict. Alternatively this effect may have been mediated by the ward team feeling valued (the health intervention focused on them personally rather than patients), however there was no mental health gain for control ward staff shown on the SF-36. Finally the gain on the control wards may have been a result of contamination from the experimental wards on the same site. Staff on the different wards were asked not to talk with each other about the interventions they were using, however there were unit managers who crossed over both wards, and the small size of many psychiatric units meant that staff often substituted for each other across wards to cover absence due to holidays or sickness, and some evidence for effects of one ward on another has been previously published (Bowers et al., 2008a,b). However, the generally low level of implementation on the experimental wards suggests that any contamination to the control wards would have been minimal. To the extent that contamination did occur, it would have diluted the experimental interventions’ effect upon the outcomes relative to the control wards, thus making the reported positive findings more robust and an underestimate of their true size.

Some of these same factors may also have led to the improvements in the Attitude to Personality Disorder Questionnaire scores on both the experimental and the control wards. However the direction of causality is open to question: better attitudes to patients might have been the result of decreased rates of difficult patient behaviour. Some previous research has indicated that this is more likely to be the case (Bowers et al., 2007a,b; Kellam et al., 1966).

It was clear that the experimental interventions had marginally greater credibility amongst the participants, with a greater number on those wards correctly able to see through the attempt to blind them. It remains therefore possible that some proportion of our positive results were due to a greater expectancy of change on the part of staff on the experimental wards.

We cannot tell from our study what the sustainability of the experimental interventions would be over the longer term, and without researcher support. The interventions have different although related targets, some to change the attitudes of staff to patients and the ways they relate to them, others to permanently extend their interactions skill set in certain common circumstances. The ten interventions were derived from the Safewards Model and were a subset of many possible additional interventions, all of which are freely available online. Clinicians may also use the underlying model to construct their own interventions. The ambition is that healthcare organisations will use the Safewards Model as a mechanism for continuing effort in improving safety and reducing coercion, and to that end Safewards has already been recommended in policy (Department of Health, 2014).

The small physical health gains occurring on the control wards were a notable and welcome outcome, but were possibly expectancy or placebo effect. The experimental interventions were not a full control for physical health interventions, as staff on the experimental wards were not primed to expect an improvement in their health as a result of their changes to practice. Nevertheless the result does suggest that there may be real occupational health value in light but efficacious health promotion interventions with staff, for which there is some other evidence (Dugdill et al., 2008).

The limitations of the Safewards trial, stated above, were the large quantity of missing data and the limited degree to which the interventions could be implemented within the short time period of the study. The study had no third arm for treatment as usual, however this would have yielded no extra benefit as the control arm of the trial controlled for the combined effect of natural change over time and the effect of participating in an experiment. Collecting data at the patient level might be seen as superior to the shift level data taken during the trial, however this would have required staff to complete at the end of every shift, in effect a matrix consisting of 22 different events and the numbers of patient on the ward concerned (on average 19), notwithstanding the problems of confidentially identifying patients in the submitted research data. Whilst this approach was considered, it was rejected as likely to lead to catastrophically low levels of data return, and a likely high degree of data errors. Placing researcher non-participant observers on the wards was also considered and rejected on two grounds. Firstly one observer only on the ward can only see what is happening where they are, thus producing a report which is not as comprehensive as that which can be collated by the whole nursing team. Secondly, cost, as an observational based trial would have required at least double the time and double the numbers of researchers. As it was, thirteen people were employed full time on the trial while it was running. It may be considered that all the items on the outcome measure (PCC) were not equally severe (e.g. a suicide attempt vs. and attempted abscond), and that some weighting method should have been used before calculating the trial outcome. However we previously conducted an expert rating exercise for the PCC, only to find that the total scores produced from actual ward data were so very highly correlated with the unweighted score as to yield no extra benefit or accuracy (Bowers et al., 2006). We therefore retained the more readily understandable unweighted scoring system. It might be argued that the study results may be biased by the presence of particularly difficult patients at different times, skewing the rates of conflict and containment on those wards. However, if such a process was occurring, we would expect any effects to be randomly spread across experimental and control wards, and different periods of the study. It is worth noting that the trial covered more than 15 years of acute ward time (31 wards × 6 months each). In addition, in other previous studies we have sought to ascertain whether individual patients can skew PCC results. In a longitudinal study collecting PCC data on sixteen wards for two years we also conducted regular interviews of staff that specifically asked about particular problem patients. It was not possible to draw a connection between particular patients and fluctuations in conflict and containment rates (Papadopoulos et al., 2012a,b).

The location of the trial in the UK may also be seen as a limitation. At the time of the trial, acute inpatient psychiatric care in the UK was composed of three main types of wards. Generic acute wards, usually serving patients of both genders, triage or assessment wards catering specifically for new admissions with a view to fast discharge, usually with higher nurse staffing levels, and psychiatric intensive care units, smaller wards with greater security and higher staffing levels for the management of more disturbed patients. Psychiatric nursing in the UK is a specialist qualification attained through a three year University based course. On average half of all ward staff are qualified nurses, the remainder unqualified healthcare assistants. These wards also benefit from input from medical staff, occupational therapists and in some cases psychologists. Safewards findings are therefore most generalisable to similar settings. Applicability and efficacy in other specialities (forensic, adolescent, or older people's psychiatric wards) and in other countries with different ward types, staffing compositions and care pathways, is therefore open to question.

In contrast to the limitations, the strengths of the Safewards trial were a theoretically generated set of interventions, advance registration, an adequately powered and credibly generalisable sample size, independent randomisation, active control for expectancy, experimental effect and change over time, independent oversight, blinding of subjects, independent and blinded statistical analysis and a demonstrable impact on conflict and containment rates.

Therefore, in the absence of any comparable quality of evidence on what makes psychiatric wards safer places, we recommend that the Safewards interventions are implemented on adult acute mental health wards, as the findings of this trial are that the gains for patients and staff may be significant. Decreased conflict means fewer injuries to patients and staff from violence, self-harm, suicide, etc., a better patient experience due to less frequent use of force and coercion by staff, including high risk procedures such as manual restraint (Paterson et al., 2003), and a significant release of staff time from dealing with conflict and containment to more positive and productive activities (Flood et al., 2008). Full instructions on how to use the Safewards interventions are freely available online, supported by instructional videos, downloadable document templates, planning and implementation guidance, and a web based forum offering support (www.safewards.net). Independent replication of the results in a further trial would make them more secure, and we recommend good quality evaluations in places where Safewards is implemented.

## Figures and Tables

**Table 1:** Baseline outcome measures.

Outcome	Experimental	Control
Primary outcomes		
PCC conflict		
Overall event rate – mean (SD)	5.22 (6.32)	4.69 (4.6)
Overall event rate – median (IQR)	3 (1–7)	4 (1–7)
Risk of events – *n*/*N*	0.80 (1391/1607)	0.87 (1293/1609)

PCC containment		
Overall event rate – mean (SD)	1.26 (1.93)	1.39 (1.94)
Overall event rate – median (IQR)	0 (0–2)	1 (0–2)
Risk of events – *n*/*N*	0.804 (938/1607)	0.866 (802/1609)

Secondary outcomes		
WAS		
Order and organisation – mean (SD)	7.19 (2.27)	6.43 (2.53)
Programme clarity – mean (SD)	7.4 (2.04)	7.18 (2.06)
Staff control – mean (SD)	1.83 (1.55)	1.8 (1.4)

SHAS		
Total – mean (SD)	78.79 (18.85)	80.16 (21.1)

APDQ		
Enjoyment – mean (SD)	4.76 (0.7)	4.8 (0.7)
Security – mean (SD)	5.11 (0.68)	5.09 (0.61)
Acceptance – mean (SD)	5.35 (0.65)	5.38 (0.56)
Purpose – mean (SD)	5.08 (0.79)	5.1 (0.85)
Enthusiasm – mean (SD)	4.28 (0.99)	4.23 (0.93)

SF-36v2		
Physical health – mean (SD)	52.19 (7.79)	51.94 (7.31)
Mental health – mean (SD)	50.24 (9.46)	50.74 (9.98)

PCC, Checklist; WAS, Ward Atmosphere Scale; SHAS, Self-Harm Antipathy Scale; APDQ, Attitudes to Personality Disorder Questionnaire. SF-36v2, Short Form 36 Health Survey v2.

**Table 2:** Estimates of treatment effects for Safewards interventions relative to control for primary and secondary outcomes.

Outcome	Estimate	95% CI	*p*-Value
Primary outcome			
PCC conflict			
Count rate ratio	0.850	0.763–0.943	0.001
Hurdle rate ratio^a^	1.139	0.915–1.426	0.234

PCC containment			
Count rate ratio	0.768	0.655–0.901	0.004
Hurdle rate ratio^a^	1.044	0.828–1.336	0.708

Secondary outcomes^b^			
WAS			
Order and organisation	−0.315	−0.792 to 0.163	0.197
Programme clarity	0.267	−0.218 to 0.753	0.281
Staff control	−0.196	−0.568 to 0.176	0.301

SHAS			
Total	0.227	−3.375 to 3.829	0.902

APDQ			
Enjoyment	0.023	−0.13 to 0.176	0.768
Security	−0.079	−0.209 to 0.05	0.231
Acceptance	0.067	−0.062 to 0.196	0.312
Purpose	−0.087	−0.28 to 0.1	0.388
Enthusiasm	0.031	−0.178 to 0.24	0.772

SF-36			
Physical health	−1.85	−3.702 to 0.003	0.05
Mental health	−0.709	−2.962 to 1.544	0.537

a Test for difference in number of zero event shifts between baseline implementation and outcome periods.

b Positive figures represent increases or improvements on the experimental wards, negative figures increases or improvements on the control wards.

**Table 3:** Number of missing shift PCC reports by trial phase and treatment condition. The number missing was the same for both conflict and containment incidents as they were on the recorded on the same report.

	Control		Treatment	
	*n*	%	*n*	%
Baseline	913	36.23	1079	40.14
Implementation	1106	43.89	1250	46.50
Outcome	1384	54.92	1524	56.70
