# Corrigendum to “Reducing conflict and containment rates on acute psychiatric wards: The Safewards cluster randomised controlled trial” [Int. J. Nurs. Stud. 52 (September (9)) (2015) 1412–1422]

**DOI:** 10.1016/j.ijnurstu.2015.12.006

**Published:** 2016-06

**Authors:** Len Bowers, Karen James, Alan Quirk, Alan Simpson, Sugar, Duncan Stewart, John Hodsoll

**Affiliations:** a Institute of Psychiatry, King's College London, De Crespigny Park, London SE5 8AF, United Kingdom; b Royal College of Psychiatrists, 21 Prescot Street, London E1 8BB, United Kingdom; c City University London, Northampton Square, London EC1V 0HB, United Kingdom

The reduction in the rate of conflict should read 15% (95% CI 5.7–23.7%) and containment 23.2% (95% CI 9.9–35.5%) in the abstract and Section 2.1. Table 1 row 8 for PCC containment, risk of events – *n*/*N* should read 0.58 (938/1607) experimental, and 0.50 (802/1609) control. In Table 2, the confidence intervals for physical health should read −3.702 to −0.003, and the corresponding intervals stated in Section 2.3 should read “The control group staff showed a 1.85 point (95% CI: −0.003, −3.704) greater improvement in physical health than the Safewards intervention”

The authors would like to apologise for any inconvenience caused.

