# Supplementary material for: Reducing conflict and containment rates on acute psychiatric wards: The Safewards cluster randomised controlled trial
Source: Int J Nurs Stud. 2015 Sep;52(9):1412–22. doi: 10.1016/j.ijnurstu.2015.05.001 (PMC4518134; doi:10.1016/j.ijnurstu.2015.05.001)
Supplement: Supplementary file 1 [file mmc1.docx]

SAFEWARDS - Supplementary Information.

**Table 4**. Rate Ratios for the different missing data scenarios for both count and hurdle parts of the models. Shown are the rate ratios with posterior mean, upper and lower 95% Bayesian Credible Intervals and the Bayesian p value.

| Model | Outcome | Posterior  Mean RR | Lower BCI | Upper BCI | p |
| --- | --- | --- | --- | --- | --- |
| **cf.20.p1** | Count | 0.812 | 0.723 | 0.914 | 0.001 |
| **cf.30.p1** | Count | 0.853 | 0.752 | 0.964 | 0.014 |
| **cf.40.p1** | Count | 0.821 | 0.718 | 0.943 | 0.004 |
| **cf.65.p1** | Count | 0.843 | 0.735 | 0.973 | 0.012 |
| **cf.mh12.1** | Count | 0.892 | 0.797 | 1.015 | 0.064 |
| **cf.mh11.1** | Count | 0.857 | 0.76 | 0.953 | 0.002 |
| **cf.mh15.1** | Count | 0.811 | 0.728 | 0.909 | 0.001 |
| **cf.3h.1** | Count | 0.866 | 0.759 | 0.983 | 0.016 |
| **cf.20.p1** | Hurdle | 1.046 | 0.852 | 1.334 | 0.73 |
| **cf.30.p1** | Hurdle | 1.07 | 0.825 | 1.399 | 0.622 |
| **cf.40.p1** | Hurdle | 1.032 | 0.761 | 1.346 | 0.846 |
| **cf.65.p1** | Hurdle | 0.851 | 0.654 | 1.163 | 0.262 |
| **cf.mh12.1** | Hurdle | 1.125 | 0.89 | 1.411 | 0.33 |
| **cf.mh11.1** | Hurdle | 1.161 | 0.935 | 1.455 | 0.198 |
| **cf.mh15.1** | Hurdle | 0.996 | 0.76 | 1.235 | 0.952 |
| **cf.3h.1** | Hurdle | 1.002 | 0.783 | 1.289 | 0.994 |
|  |  |  |  |  |  |
|  |  |  |  |  |  |
| **ct.20.p1** | Count | 0.747 | 0.634 | 0.894 | 0.001 |
| **ct.30.p1** | Count | 0.756 | 0.61 | 0.919 | 0.004 |
| **ct.40.p1** | Count | 0.708 | 0.552 | 0.877 | 0.008 |
| **ct.65.p1** | Count | 0.725 | 0.603 | 0.877 | 0.001 |
| **ct.mh12.1** | Count | 0.759 | 0.645 | 0.907 | 0.002 |
| **ct.mh11.1** | Count | 0.743 | 0.627 | 0.888 | 0.002 |
| **ct.mh15.1** | Count | 0.747 | 0.637 | 0.892 | 0.001 |
| **ct.3h.1** | Count | 0.704 | 0.59 | 0.866 | 0.001 |
| **ct.20.p1** | Hurdle | 1.099 | 0.872 | 1.393 | 0.432 |
| **ct.30.p1** | Hurdle | 1.073 | 0.847 | 1.4 | 0.552 |
| **ct.40.p1** | Hurdle | 0.866 | 0.638 | 1.166 | 0.392 |
| **ct.65.p1** | Hurdle | 0.958 | 0.727 | 1.284 | 0.764 |
| **ct.mh12.1** | Hurdle | 1.017 | 0.8 | 1.289 | 0.854 |
| **ct.mh11.1** | Hurdle | 1.047 | 0.834 | 1.3 | 0.744 |
| **ct.mh15.1** | Hurdle | 1.041 | 0.839 | 1.312 | 0.748 |
| **ct.3h.1** | Hurdle | 1.025 | 0.784 | 1.311 | 0.84 |
|  |  |  |  |  |  |

**Key to model names in Table 4.** Conflict: Model codes and biases introduced. In this table the codes refer to analysis models for conflict events. For containment events the codes were identical apart from the prefix being ct rather than cf.

|  | **Model Code** | **Assumption** |
| --- | --- | --- |
| **Ward Exclusions** | |  |
|  | **cf.20.p1** | Wards returning less than 20% of shift reports (PCC) |
|  | **cf.30.p1** | Wards returning less than 30% of shift reports (PCC) |
|  | **cf.40.p1** | Wards returning less than 40% of shift reports (PCC) |
|  | **cf.65.p1** | Wards returning more than 65% of shift reports (PCC) |
|  | | |
|  | **cf.mh12.1** | Hospital 12 for Ward 4 – problems following protocol |
|  | **cf.mh11.1** | Hospital 11 for Ward 24 – problems following protocol |
|  | **cf.mh15.1** | Hospital 15 for Ward 25 – very high event rates |
|  | **cf.3h.1** | All 3 hospitals: 12, 11 and 15 |

**Table 5.** Rate ratios for effect of treatment for imputed data sets in which biases have been introduced into all missing observations. See Table 2 above and SI text. Shown are the rate ratios with posterior mean, upper and lower 95% Bayesian Credible Intervals and the Bayesian p value.

| **Model** | **Outcome** | **Posterior**  **Mean** | **Lower BCI** | **Upper BCI** | **p** |
| --- | --- | --- | --- | --- | --- |
| **Conflict** |  |  |  |  |  |
| **cf.rs0a.1** | Count | 0.851 | 0.763 | 0.947 | 0.006 |
| **cf.rs.p1.c6** | Count | 0.903 | 0.857 | 0.952 | 0.001 |
| **cf.rs.p1.c50** | Count | 0.895 | 0.842 | 0.946 | 0.001 |
| **cf.rs.p1.c100** | Count | 0.861 | 0.741 | 0.994 | 0.038 |
| **cf.rs0a.1** | Hurdle | 1.126 | 0.946 | 1.293 | 0.162 |
| **cf.rs.p1.c6** | Hurdle | 1.098 | 0.913 | 1.346 | 0.362 |
| **cf.rs.p1.c50** | Hurdle | 1.088 | 0.912 | 1.295 | 0.364 |
| **cf.rs.p1.c100** | Hurdle | 1.085 | 0.917 | 1.339 | 0.416 |
|  |  |  |  |  |  |
| **Containment** |  |  |  |  |  |
| **ct.rs0a.1** | Count | 0.766 | 0.643 | 0.899 | 0.002 |
| **ct.rs.p1.c2** | Count | 0.83 | 0.746 | 0.928 | 0.001 |
| **ct.rs.p1.c50** | Count | 0.902 | 0.841 | 0.964 | 0.006 |
| **ct.rs.p1.c100** | Count | 0.844 | 0.712 | 1.046 | 0.084 |
| **ct.rs0a.1** | Hurdle | 1.014 | 0.865 | 1.239 | 0.912 |
| **ct.rs.p1.c2** | Hurdle | 0.939 | 0.8 | 1.081 | 0.444 |
| **ct.rs.p1.c50** | Hurdle | 0.969 | 0.823 | 1.123 | 0.706 |
| **ct.rs.p1.c100** | Hurdle | 0.965 | 0.838 | 1.151 | 0.628 |
|  |  |  |  |  |  |

**Key to model names in Table 5.** Sensitivity analysis to see effect of assuming missing observations have either 0 or more events than average. For the first case, each missing observation was filled in as a zero. For the second case, missing values were substituted by hot-deck imputation of observed data which had overall mean of event rate added to ensure events were greater than mean.

|  | | **Shifts with events** | **Hurdle (p of events)** |
| --- | --- | --- | --- |
|  | **cf.rs0a.1** | + 0 (0%) | 0 |
|  | **cf.rs.p1.c6** | + 6 shift events (100%) | 1 |
|  | **cf.rs.p1.c50** | + 50 shift events (1000%) | 1 |
|  | **cf.rs.p1.c100** | + 100 shift events (2000%) | 1 |
|  |  |  |  |
|  | **ct.rs0a.1** | + 0 (0%) | 0 |
|  | **ct.rs.p1.c6** | + 2 shift events (100%) | 1 |
|  | **cf.rs.p1.c50** | + 50 shift events (2500%) | 1 |
|  | **cf.rs.p1.c100** | + 100 shift events (5000%) | 1 |
|  |  |  |  |

**Table 6.** Sensitivity analysis in which imputed observations were biased in either the experimental or control arm, see Table 3 and SI text. Shown are the rate ratios, upper and lower 95% Bayesian Credible Intervals and the Bayesian p value.

| **Model** | **Bias Arm** | **Outcome** | **Posterior**  **Mean** | **Lower BCI** | **Upper BCI** | **p** |
| --- | --- | --- | --- | --- | --- | --- |
|  |  |  |  |  |  |  |
| **cf3.rs.122** | **Experiment** | **Count** | 1.014 | 0.927 | 1.113 | 0.74 |
| **cf3.rs.122** |  | **Hurdle** | 1.018 | 0.837 | 1.24 | 0.82 |
|  |  |  |  |  |  |  |
| **cf3.rs.121** | **Control** | **Count** | 0.738 | 0.671 | 0.805 | 0.001 |
| **cf3.rs.121** |  | **Hurdle** | 1.03 | 0.849 | 1.252 | 0.776 |
|  |  |  |  |  |  |  |
| **ct3.rs.112** | **Experiment** | **Count** | 1.019 | 0.878 | 1.163 | 0.814 |
| **ct3.rs.112** |  | **Hurdle** | 1.047 | 0.855 | 1.277 | 0.626 |
|  |  |  |  |  |  |  |
| **ct3.rs.111** | **Control** | **Count** | 0.686 | 0.597 | 0.783 | 0.001 |
| **ct3.rs.111** |  | **Hurdle** | 1.08 | 0.889 | 1.319 | 0.45 |
|  |  |  |  |  |  |  |

**Key to model names in Table 6.** Add biases selective to either experimental or control arm to hot-deck imputation of observations. For each arm, the number of imputed observations is the difference in rates for each phase relative to the baseline rate of missing data.

| **Bias** | **Model** | **Shifts with events** | **Hurdle (p of events)** |
| --- | --- | --- | --- |
| **Conflict** |  |  |  |
| **Experiment** | **cf3.rs.122** | + 2 (33% of mean) | 1 |
| **Control** | **cf3.rs.121** | + 2 (33% of mean) | 1 |
| **Containment** |  |  |  |
| **Experiment** | **ct3.rs.121** | + 1 (70% of mean) | 1 |
| **Control** | **ct3.rs.111** | + 1 (70% of mean) | 1 |
|  |  |  |  |
